# Tregitopes Improve Asthma by Promoting Highly Suppressive and Antigen-Specific Tregs

**DOI:** 10.3389/fimmu.2021.634509

**Published:** 2021-04-19

**Authors:** Marieme Dembele, Shao Tao, Amir H. Massoud, S. M. Shahjahan Miah, Sandra Lelias, Anne S. De Groot, Bruce D. Mazer

**Affiliations:** ^1^ The Research Institute of the McGill University Health Centre, Division of Pediatric Allergy Immunology and Dermatology, Montreal Children’s Hospital, McGill University Health Centre, Montreal, QC, Canada; ^2^ EpiVax, Inc., Providence, RI, United States; ^3^ Center for Vaccines and Immunology, College of Veterinary Medicine, University of Georgia, Athens, GA, United States

**Keywords:** Tregitope, allergic airways disease (AAD), adoptive transfer, T regulatory cells (Tregs), induced Tregs (iTregs), murine model of asthma, allergic asthma

## Abstract

Tregitopes (T regulatory epitopes) are IgG-derived peptides with high affinity to major histocompatibility complex class II (MHCII), that are known to promote tolerance by activating T regulatory cell (Treg) activity. Here we characterized the effect of IgG Tregitopes in a well-established murine model of allergic asthma, demonstrating *in vivo* antigen-specific tolerance *via* adoptive transfer of Tregitope-and-allergen-activated Tregs. Asthma is a heterogeneous chronic inflammatory condition affecting the airways and impacting over 300 million individuals worldwide. Treatment is suppressive, and no current therapy addresses immune regulation in severely affected asthmatics. Although high dose intra-venous immunoglobulin (IVIg) is not commonly used in the asthma clinic setting, it has been shown to improve severe asthma in children and in adults. In our laboratory, we previously demonstrated that IVIg abrogates airway hyperresponsiveness (AHR) in a murine model of asthma and induces suppressive antigen-specific T-regulatory cells. We hypothesized that IgG-derived Tregitopes would modulate allergic airway disease by inducing highly suppressive antigen-specific Tregs capable of diminishing T effector cell responses and establishing antigen-specific tolerance. Using ovalbumin (OVA-) and ragweed-driven murine models of allergic airway disease, we characterized the immunoregulatory properties of Tregitopes and performed Treg adoptive transfer to OVA- and ragweed-allergic mice to test for allergen specificity. Treatment with Tregitopes attenuated allergen-induced airway hyperresponsiveness and lung inflammation. We demonstrated that Tregitopes induce highly suppressive allergen-specific Tregs. The tolerogenic action of IgG Tregitopes in our model is very similar to that of IVIg, so we foresee that IgG Tregitopes could potentially replace steroid-based treatment and can offer a synthetic alternative to IVIg in a range of inflammatory and allergic conditions.

## Introduction

Asthma is a heterogeneous chronic inflammatory condition affecting the airways. The global incidence of asthma is on the rise, with more than 300 million individuals diagnosed worldwide. It is also the most prevalent chronic disease in children globally, causing significant morbidity in adults, youth, and children (1). There are many triggers for asthma symptoms, including allergens, infectious agents and environmental factors, including pollution. The most common predisposition for asthma is a history of atopy, noted in up to 70% of children and 50% of adults.

There are several existing endotypes of asthma, and specific inflammatory profiles characterize each subtype. Current treatment strategies include bronchodilators, corticosteroids and, for severe asthma, biological therapies, such as anti-IgE and anti-IL5 (2). However, given the heterogeneity and complexity of the disease, finding effective treatments applicable to all subtypes is challenging. While most novel treatments are aimed at ameliorating the inflammatory component of asthma by targeting a single pathway, treatments that can better regulate the inflammatory response may be more successful, with less secondary effects.

Our laboratory has previously demonstrated that intravenous immunoglobulin G (IVIg) attenuates murine allergic airways disease (AAD) by inducing highly suppressive T-regulatory cells (Treg) (3, 4). Most recently, we have established that the Treg induced by IVIg are antigen-specific (5). T regulatory cell epitopes (Tregitopes) potentially offer a synthetic alternative to IVIg (6). Tregitopes are peptides derived from conserved sequences in the Fc and Fab region of IgG, which bind with moderate to high affinity to multiple major histocompatibility complex class II (MHCII) molecules, for subsequent presentation to T cells (7). Rather than activating T effector immune responses, these highly conserved, HLA DRB1-promiscuous T cell epitopes appear to activate natural regulatory T cells.

Since their identification in 2008 by De Groot and Martin, a wide range of mouse studies have explored the anti-inflammatory properties of IgG Tregitopes in a range of pre-clinical models. Potential applications that have been evaluated include Tregitope treatment for Type I diabetes (8), cockroach induced-allergy  (9), experimental colitis (10) and pregnancy loss (11). Several studies have demonstrated Tregitope’s ability to induce tolerance by activating and expanding Treg *in vitro* and *in vivo*. Furthermore, many studies show a local and systemic increase in transcription and translation of immunosuppressive cytokines such as TGFβ and IL-10 upon treatment with IgG Tregitopes (9, 11).

Mechanistic commonalities with IVIg, including Treg induction and anti-inflammatory characteristics, prompted us to explore the suppressive capacity and antigen-specificity of Treg in murine models of allergen induced asthma. We hypothesized that IgG Tregitopes would abrogate allergic airway disease by inducing highly suppressive T regulatory cells capable of modulating (or modifying) T effector cells and would lead to the induction of tolerance in an antigen-specific manner. We demonstrate that IgG Tregitopes indeed diminish allergic inflammation in murine lungs and can induce highly suppressive allergen-specific Treg.

## Materials and Methods

### Animals

C57BL/6 (WT), B6.Cg-*Foxp3^tm2Tch^*/J (B6-Foxp3^EGFP^) and B6.Cg-Tg(TcraTcrb)425Cbn/J (OT-II) mice were acquired from the Jackson Laboratories and bred in a pathogen-free environment in the animal facility of the Research Institute of the McGill University Health Center. 6 to 10 week old mice were used in the experiments. All protocols were approved by the McGill University Animal Care Committee.

### Generation of Bone Marrow Derived Dendritic Cells

Bone-marrow was extracted from the femur and tibia of CO2 euthanized WT mice and cultured at a concentration of 0.5 million/ml in 6 well plates in complete medium supplemented with 20ng/ml granulocyte-macrophage colony-stimulating factor (GMCSF, Peprotech). Fresh medium was added on days 3, 5, 7, and 9. BMDCs were harvested in a two-step process. First, non-adherent cells contained in media were removed by aspiration. Second, loosely adherent cells were harvested by adding 2 ml of ice-cold media in each well and gently shaking the plate at 4°C for 10 minutes. After centrifugation, both non-adherent and loosely adherent cells were mixed and resuspended in fresh medium. This suspension was composed of 90% CD11c+ expressing cells. Cells were used for experiments on Day 10 of culture.

### Ovalbumin-Induced Allergic Airway Disease Model

WT Mice were sensitized, intraperitoneally (IP), with 50μg of ovalbumin (OVA, Sigma-Aldrich) and 800 μg of aluminum hydroxide (AlOH, Sigma-Aldrich) in 200 μl of sterile phosphate buffered saline (PBS, Wisent Bioproducts). IP injection of OVA/AlOH was performed on days 0 and 14. On day 27, mice were treated IP with IVIg (2g/kg, Hema Quebec), vehicle control (<0.5% DMSO in PBS) and two different mixtures of Tregitopes (21^st^ Century Biochemicals) emulsified in 500 μl sterile PBS. Two mixtures of human Tregitopes (hTR), mixture hTR 084/289 (25μg hTregitope 084 + 25 μg hTregitope 289) and mixture hTR 167/289 (25μg hTregitope 167 + 25 μg hTregitope 289), and a range of concentrations of murine Tregitope, mTR 167 (mTregitope167) as defined in earlier dosing studies, were prepared in PBS solution with a final DMSO concentration of <0.5%. Human Tregitopes have been demonstrated to bind to murine MHC and were used in these experiments to enable subsequent translation to human studies (12).

From day 28 through 30, mice were challenged intranasally with 20μl of a 1% OVA preparation. Alternatively, for the adoptive transfer experiments, bone marrow-derived dendritic cells (BMDCs) were stimulated overnight with 300 μg/ml OVA *ex vivo*. Cells were washed twice and 1 million BMDCs in 100μl PBS were transferred intratracheally (IT) in each lung. On day 10, mice were challenged with 50 ml of a 1.5% OVA preparation and euthanized 72 hours later.

### Ragweed-Induced Allergic Airway Disease Model

WT Mice were sensitized IP on days 0 and 4 with a 100 μl solution composed of 75 μl 1 mg/ml of mixed ragweed (Greer Labs) and 25 μl of Imject aluminum adjuvant (Thermo Fisher Scientific) in sterile PBS as previously described (5). On day 10, mice were treated IP with vehicle control, IVIg or hTregitopes. From day 11 through 13, mice were challenged intranasally with 20 μl of a 0.1% ragweed. Alternatively, day-10 bone marrow-derived dendritic cells (BMDCs) were stimulated overnight with 100 μg/ml ragweed. Cells were washed twice and 1 million BMDCs in 100μl PBS were transferred in each lung IT. On day 10, mice were challenged IT with 50 μl of a 1.5% ragweed preparation and euthanized 72 hours later.

### Preparation of Lung, Spleen, and Lymph Node Cell Suspensions

Lung perfusion was performed by injecting 10 ml of cold PBS through the right ventricle. Harvested lungs were collected in a solution containing Collagenase D (0.15 U/ml, Roche Life Sciences) and DNase (40U/ml, Roche Life Science) dissolved in a Hank’s Balanced Salt Solution (HBSS) supplemented with calcium and magnesium (Thermo Fisher). Lungs were subsequently placed in Gentle MACs C tubes and processed using the GentleMACS Dissociator (Miltenyi Bio-tec) for 30 minutes at 37°C. The cell suspension was filtered through a 40mm strainer and lysed using red blood cell lysis solution (RBC Lysis Buffer, Biolegend). Cells were washed twice with PBS, counted and used for assays. Spleen and mediastinal lymph nodes were collected in PBS and passed through a 40mm cell strainer. After lysis, cells were reconstituted in PBS for further use.

### Lung Homogenates

Lungs were harvested and placed in cold RPMI. Gentle MACS M tubes (Miltenyi Biotec) were used to homogenize the tissue, and supernatant was collected and stored at -80°C for cytokine analysis.

### Lung Histology

Lungs were fixed by inflation with 1 ml of 10% formalin and processed. Sections of 0.5mm were stained with H&E and scanned using the digital scanner Aperio AT Turbo. For scoring, peri-bronchial inflammation and the quality of infiltrates were assessed and added using the following grading system: none = 0, mild = 1, moderate = 2, severe = 3 (13).

### Cytokine ELISA

Total serum IgE, and levels of IFNγ, IL-13, IL-17 in lung homogenates were measured using commercial ELISA kits (Biolegend). OVA-specific IgE was measured as previously reported (5).

### Flow Cytometry

Flow cytometry analysis was performed using a Becton-Dickinson LSR Fortessa 20X or CANTO II. We used the following antibodies: CD4 (clone RM4-5, BV510 or FITC, Biolegend), CD25 (3C7, PE, Biolegend or PC61, BV605, Biolegend), Siglec-F (LOU, BV421, BD Biosciences), Foxp3 (FJK-16s, APC, eBioscience), Helios (22F6, Pacific blue, Biolegend), CD11c (N418, APC, Biolegend), MHCII (M5/114.15.2, BV510, Biolegend), CD11b (M1/70, PercP, Biolegend), Ly6G (1A8, PE/Cy7, Biolegend), Ly6C (HK1.4, PE, Biolegend), IL-13 (eBio13A, PE, eBioscience), IL-17A (TC11-18H10.1, PercP/Cy5.5 or AlexaFluor700, Biolegend), IFNγ (4S.B3, BV421 or XMG1.2, APC/Cy7, Biolegend), CD45 (30-F11, Alexa Fluor700 or APC, Biolegend), fixable viability dye (Zombie Aqua, Biolegend), and fixable viability dye (eFluor780, eBioscience).

### Treg and Naïve CD4+ T Cell Purification

CD4+ T cells were isolated from cell suspensions of lung, LNs and spleen from Foxp3^EGFP^ mice using the EasySep CD4+ T cell isolation kit (Stemcell). Subsequently, cells were stained for CD25, CD4 and viability dye, and CD25+Foxp3+ cells were sorted using FACS ARIA II (Becton Dickinson). For *in vitro* OTII studies, naïve syngeneic CD4+ T cells and CD25+ Tregs were isolated using the mouse EasySep naïve and Treg isolation kits, respectively (Stemcell). For adoptive transfer experiments, isolated Tregs were administered IT (50,000/mouse), one day prior to challenge with OVA or ragweed.

### Treg Suppression Assay

To study the suppressive effect of Treg induced by human Tregitopes on murine CD4+ effector T function, naïve OTII-CD4+ responder cells were stained with 1μM of CFSE at 37°C for 7 minutes. CD25+ Tregs were purified from lung, spleen and LNs of Control- and Tregitope- treated OVA-allergic mice. OTII-CD4+ responder cells were then co-cultured with either Control- or Tregitope- induced CD25+ Tregs in the presence of OVA-pulsed CD11c+ cells in a 96 wells round-bottom plate at 37°C. CFSE staining was assessed by flow cytometry after 96 hours of culture.

### Airway Hyperresponsiveness Measurement

Airway hyperresponsiveness (AHR) to methacholine was measured, as previously described by Massoud et al. (3, 5) using Flexivent for small animals (SCIREQ, Montreal, Canada).

### Statistical Analysis

Statistical analysis was carried out using GraphPad Prism 6 (GraphPad). One- or two- way ANOVA test was used for statistical analysis. Statistical significance was considered to be achieved when p < 0.05. All graphed data represent at least 2 independent experiments.

## Results

### Tregitopes Improve AHR in an OVA-Induced Murine Allergic Airways Disease Model

**Figure 1:**
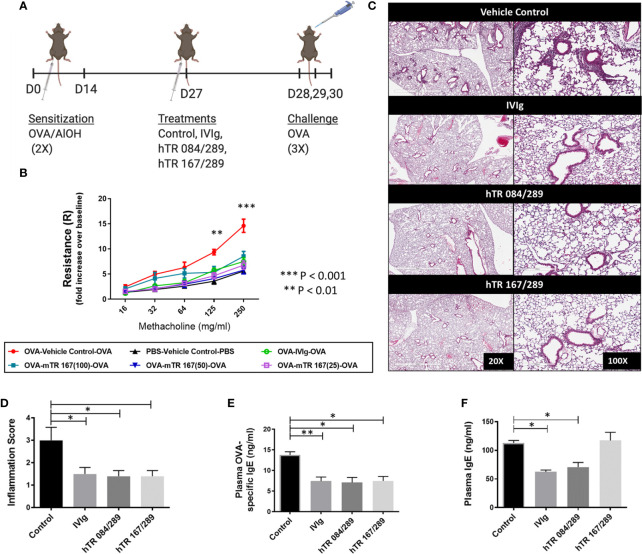
Tregitopes improve OVA-driven murine allergic airways disease. When compared to IVIG, IgG Tregitopes were equally as effective at reducing airway disease. **(A)** Timeline of the OVA-driven model of murine allergic airways disease used in this experiment. Sensitization to OVA/AlOH was performed on days 0 and 14. Treatment with Control, IVIg and Tregitopes was administered on day 27. Challenge was done intranasally with OVA on days 28, 29 and 30. **(B)** Airway hyperresponsiveness to methacholine was performed on day 31 (2 independent experiments, n = 3). **(C)** Representative images of formalin-fixed lung slides stained with H&E and **(D)** associated inflammation scores for the Control, IVIg, hTR 084/289 and hTR 167/289 treatment groups (2 independent experiments, n = 4-5). Two pathologists, blinded to the study, assessed and graded the slides for peri-bronchial inflammation and quality of cell infiltrates. For each group 8-10 slides from 4-5 animals were used in the evaluation. **(E)** OVA-specific IgE in plasma and **(F)** Total IgE, measured by ELISA. ***p, 0.005, **p, 0.01, *p, 0.05, one-way ANOVA test.

We have previously demonstrated that intravenous immunoglobulin G (IVIg) treatment attenuates lung inflammation and AHR in an OVA-driven model of AAD (3, 5, 14, 15). Initially we evaluated whether a single mTregitope167 (mTR 167) could replicate the therapeutic effect of IVIg in this model (**Figure 1A**). This dose-response study with mTR 167 assessed the impact of Tregitope treatment on AHR improvement. OVA sensitization and challenge significantly increased airway resistance, as measured by Flexivent, in mice treated with the vehicle control for Tregitopes. In contrast, AHR was diminished by treatment with IVIg (**Figure 1B**) and also by mTregitope 167 at all three doses in this dose-ranging study (25μg/ml, 50μg/ml, 100μg/ml) (**Figure 1B**). In this study, mTregitope 167 was as effective as IVIg, with treatment resulting in airway resistance measures that were comparable to the non-sensitized PBS group (**Figure 1B**).

As we had confirmed that human Tregitopes (hTR) bind equally well to human MHC and to MHC class II of C57B/6 mice (I-Ab) and were as effective as murine Tregitopes (mTR) in murine studies *in vivo* (12), the remaining assays were performed with hTR. **Supplemental Table S1** lists the sequences and EpiMatrix binding Z-scores for all of the Tregitopes used in these studies. To explore the potential synergy of multiple Tregitopes, we used two combinations of human Tregitopes: hTregitope 289 with hTregitope 084 (hTR 084/289) and hTregitopes 289 with hTregitope 167 (hTR 167/289) at a total concentration of 50μg/ml (25 μg per Tregitope) for each mixture.

### Tregitope Treatment Abrogates Airway Inflammation and Diminishes Inflammatory Cytokine Production

H&E stained lung sections from Tregitope-treated mice demonstrated reduced peri-vascular and peri-bronchial infiltrates compared to the vehicle control group (**Figure 1C**). The inflammation score was also significantly reduced in Tregitope-treated groups (**Figure 1D**). As expected, there was also a significant reduction in OVA-specific IgE, as measured by ELISA in both Tregitope groups (**Figure 1E**). Total IgE, however, was only reduced in the hTR 084/289 group (**Figure 1F**).

**Figure 2:**
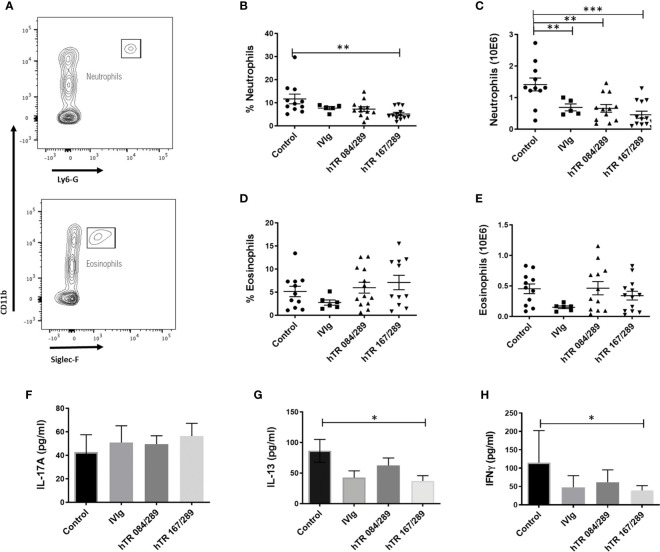
Tregitopes modulate neutrophil and cytokine profiles of mice in OVA-driven murine allergic airways disease. Neutrophils and IL-13 are significantly reduced in the lungs of mice treated with IVIG or Tregitopes. **(A)** Gating strategy for lung neutrophils and eosinophils of OVA-allergic mice undergoing treatment with Control, IVIg, or hTR 084/289 and hTR 167/289. **(B)** Percentage and **(C)** absolute number of lung neutrophils. **(D)** Percentage and **(E)** absolute number of lung eosinophils (representative of 3 independent experiments, n = 5-13). **(F)** Cytokines concentration of IL-17A **(G)** IL-13 and **(H)** IFNγ measured by ELISA in whole lung homogenates. ***p, 0.005, **p, 0.01, *p, 0.05, one-way ANOVA test.

We further investigated the effect of Tregitopes on lung inflammation by performing cellular phenotyping of lung eosinophils and neutrophils and by measuring cytokines IL-17A, IL-13 and IFNγ in whole lung extracts. Neutrophils were detected in the Ly6G+CD11b+ gate while eosinophils were defined as Ly6G-SiglecF+CD11b+CD11c- (**Figure 2A**). Our model was neutrophil-dominant, and neutrophilia was significantly diminished, in terms of percentage and total cell number, upon both IVIg and Tregitope treatment (**Figures 2B, C** **)**. The percentage of lung eosinophils was not significantly reduced, while total eosinophils tended to be lower (albeit not significantly) in the IVIg and hTR 167/289 treatment groups as compared to vehicle control (**Figures 2D, E** **)**. Although IL-17A levels of total lung homogenates remained constant (**Figure 2F**), IL-13 decreased in the lung homogenates of all OVA-Tregitope treatment groups and significantly in the hTR 167/289 group as compared to control (**Figure 2G**). IL-17A is a proinflammatory cytokine produced by innate (iNKT, NKT, γδTcells, mast cells, neutrophils) as well as adaptive (IL17-A producing CD4+ T cells) immune cells (16–18). IL-17A production by innate cells occurs within a few hours post-antigen or pathogen exposure while Th17 cells differentiation is a process that takes days. At the study time point, we are capturing accumulation of IL-17A cytokine by all cell types, over several days, in total lung. It is possible that an earlier time point might show a different IL-17A distribution across groups in whole lung.

IFNγ decreased across all three treatment groups (**Figure 2H**) with significance for hTR 167/289. Again, this is probably due to the level of IFNγ accumulation over time after treatment with Tregitopes, or Tregitope effect may be Th2 biased and have a greater effect on other cytokines. A time course analysis of cytokine changes over time would address this issue.

### Tregitopes Ameliorate Ragweed-Driven Murine Allergic Airways Disease

**Figure 3:**
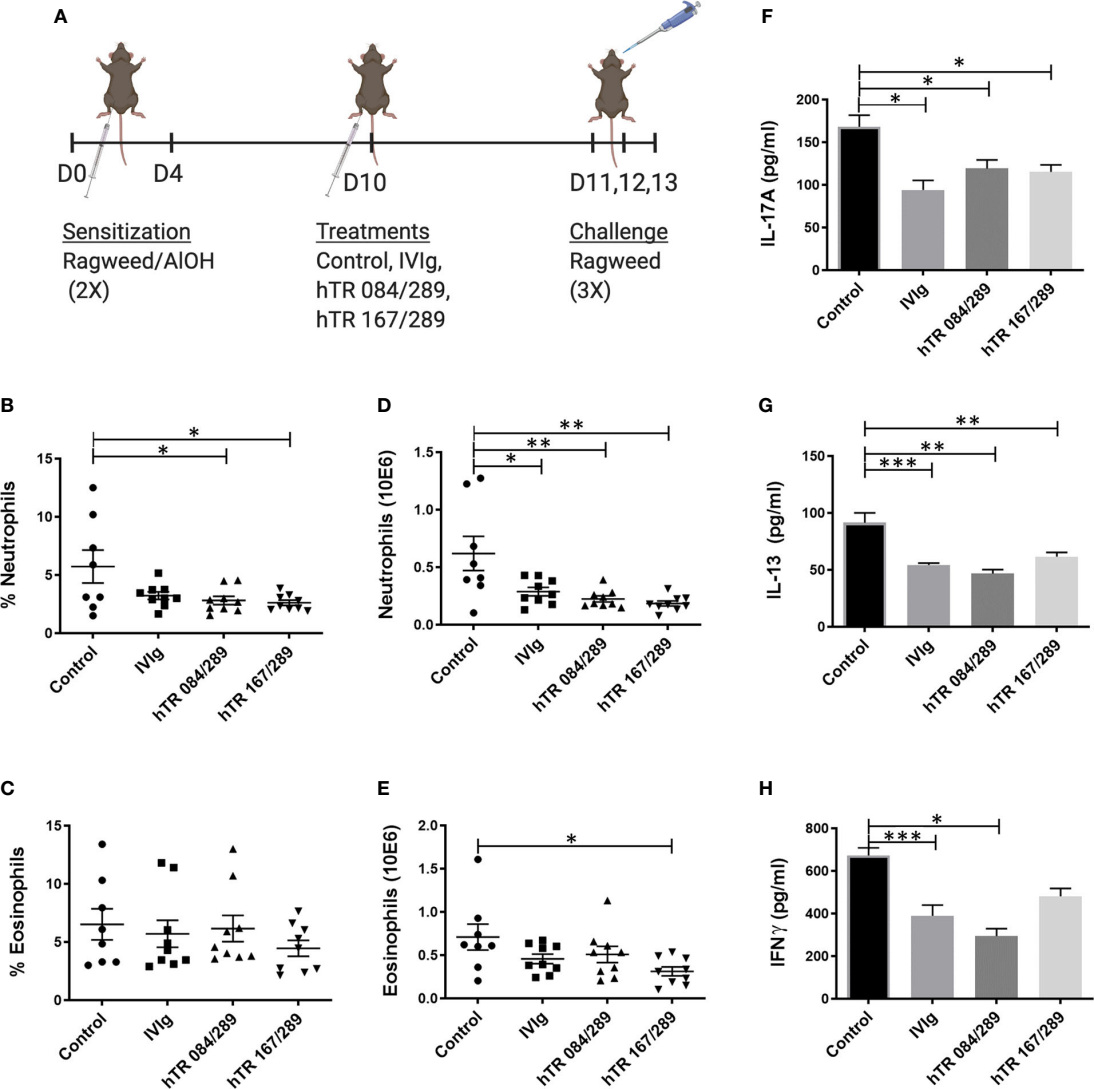
Tregitopes modulate lung granulocytes and cytokines profile of mice with ragweed-driven murine allergic airways disease. In the more acute ragweed model of allergic airway diseases, Tregitopes (and IVIG) significantly reduce cellular infiltration (neutrophil as well as eosinophils) and broadly reduce cytokine levels in the lungs. **(A)** Timeline of the ragweed-driven model of murine allergic airways disease used in this experiment. Sensitization to OVA/AlOH was performed on days 0 and 4. Treatment with Control, IVIg or Tregitopes was administered on day 10 and challenge was done intranasally with ragweed on days 11,12 and 13. **(B)** Percentage and **(D)** absolute number of neutrophils. **(C)** Percentage and **(E)** absolute number of lung eosinophils in the lungs of ragweed-allergic mice undergoing treatment with Control, IVIg or Tregitopes on day 10 (representative of 3 independent experiments, n = 5-9). **(F)** Cytokines concentration of IL-17A, **(G)** IL-13 and **(H)** IFNγ measured by ELISA in whole lung homogenates. (representative of 3 independent experiments, n = 5-9). ***p, 0.001, **p, 0.01, *p, 0.05, one-way ANOVA test.

We further tested the immunomodulatory properties of IVIg and Tregitopes in the ragweed-driven model of murine AAD. We used a systemic 14-day acute model, which does not elicit significant airway remodeling (data not shown) (**Figure 3A**). In this model, lung neutrophilia and eosinophilia were similarly prevalent both in terms of cell frequency and total cell number. Treatment with IVIg and Tregitopes significantly reduced the proportion of lung neutrophils as compared to other cells and the total cell count of lung neutrophils (**Figures 3B, D**). There was a trend for the percentage of lung eosinophilia to decrease (albeit not significantly) (**Figure 3C**), however, there was a significant decrease in absolute eosinophil count following treatment with hTR 167/289 (**Figure 3E**).

Similar to the observations made with OVA-Tregitope treatment (**Figure 2**), lung cytokines decreased in ragweed-Tregitope-treated animals. IL-17A, IL-13 and IFNγ levels in whole lung homogenates were markedly lower in mice treated with IVIg, hTR 167/289, and hTR 084/289 (**Figures 3F–H**).

### Tregitopes Modulate Effector T Cell Responses

We assessed Th1, Th2, and Th17 effector T cell responses by measuring intracellular IFNγ, IL-13, and IL-17A, respectively, in PMA/ionomycin stimulated lung cells from allergic mice (Tregitopes treated and vehicle control) (See **Supplemental Figure S1**). Unstimulated cells were used to establish cytokine gating. While IFNγ production by CD4+ cells did not decrease following treatment with Tregitopes in the OVA and ragweed models, in contrast, IL-17A producing cells were significantly decreased upon treatment with both Tregitope mixtures. In the OVA model, IL-13 expression in CD4+T cells and in total cells was significantly reduced in the Tregitope treatment group as in the ragweed model. Taken together, Th2 and Th17 responses in AAD were attenuated by treatment with hTregitopes.

### Tregitopes Induce Highly Suppressive Antigen-Specific Tregs

Tregs are important immunoregulatory cells in asthma. We have shown that IVIg promotes generation and activation of CD25highFoxp3+ peripheral Tregs (pTregs) and increases the suppressive function of the pTregs (5, 15). We hypothesized that pTregs from Tregitope-treated mice would be more suppressive than Tregs from the vehicle-treated control group *in vivo* and *in vitro*.

**Figure 4:**
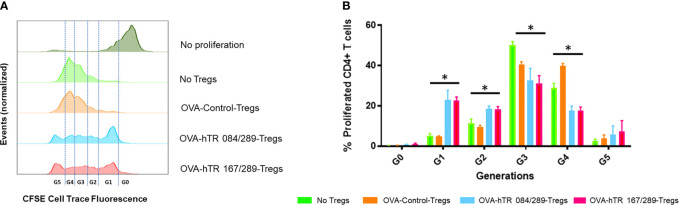
Tregitopes induce highly suppressive Tregs in an *in vitro* bystander assay. An OVA-specific co-culture system was established by co-culturing OVA-specific OTII-CD4+ responder T cells, OVA-stimulated splenic DCs from OTII mice, and Tregs isolated from OVA-allergic mice treated with Control, hTR 084/289 or hTR 167/289. Proliferation was monitored by cell tracer staining and flow cytometry. **(A)** Representative histograms of cell tracer stained CD4+ proliferating cells and **(B)** frequency of CD4+ proliferating cells associated with generational distribution after 4 days of co-culture. *p, 0.05, one-way ANOVA test.

Here we have tested the effect of Tregitope treatment in the induction of Tregs in ragweed-allergic mice. We observed that the frequency of CD25highFoxp3+ pTregs and Helios+Foxp3+ pTregs in mediastinal lymph nodes of ragweed-allergic mice was augmented upon Tregitope treatment compared to vehicle treatment (**Supplemental Figures S3A–C**). We subsequently tested the suppressive ability of Tregs derived from allergic mice treated with Control or Tregitopes, *in vitro*, using an OVA-specific suppression assay. We therefore isolated CD25+Foxp3+ Tregs from spleen, lymph nodes, and lungs from differentially treated OVA-allergic mice. We tested the ability of these Tregs to suppress OVA-specific OTII+ CD4+ T cells proliferation *in vitro* using an OVA-specific suppression assay at a 16:1 ratio of OVA-specific CD4+ T cells (derived from OTII TCR transgenic mice (15)) to Treg cells isolated from OVA- and Tregitope-treated allergic mice. The Tregitope-activated (OVA-hTR 084/289 and OVA-hTR 167/289)-Tregs were significantly more suppressive than OVA-Control-Tregs (**Figures 4A, B** **)**. Both sets of Tregitope-treated (OVA-hTR 084/289-Tregs and OVA-hTR 167/289)-Tregs also demonstrated higher suppressive capacity than OVA-Control-Tregs groups at all ratios (**Figures 4A, B**). This experiment corroborates findings from an earlier study that was performed using OVA in ex vivo cultures of CD4+ T cells from Tregitope-treated DO11.10 mice (19).

### Tregitopes Induce Antigen-Specific Tregs

Inducing an antigen-specific regulatory response is essential for successful treatment strategies in allergic asthma. Since we had previously established that IVIg elicits tolerance by inducing peripheral antigen-specific Tregs (5), we wished to determine whether the Tregitope- and allergen-treated Tregs would demonstrate antigen-specificity in the same model. This experiment involved inducing allergen-specific Tregs to either OVA or Ragweed, and then transferring the Tregs into the lungs of OVA-sensitized mice.

**Figure 5:**
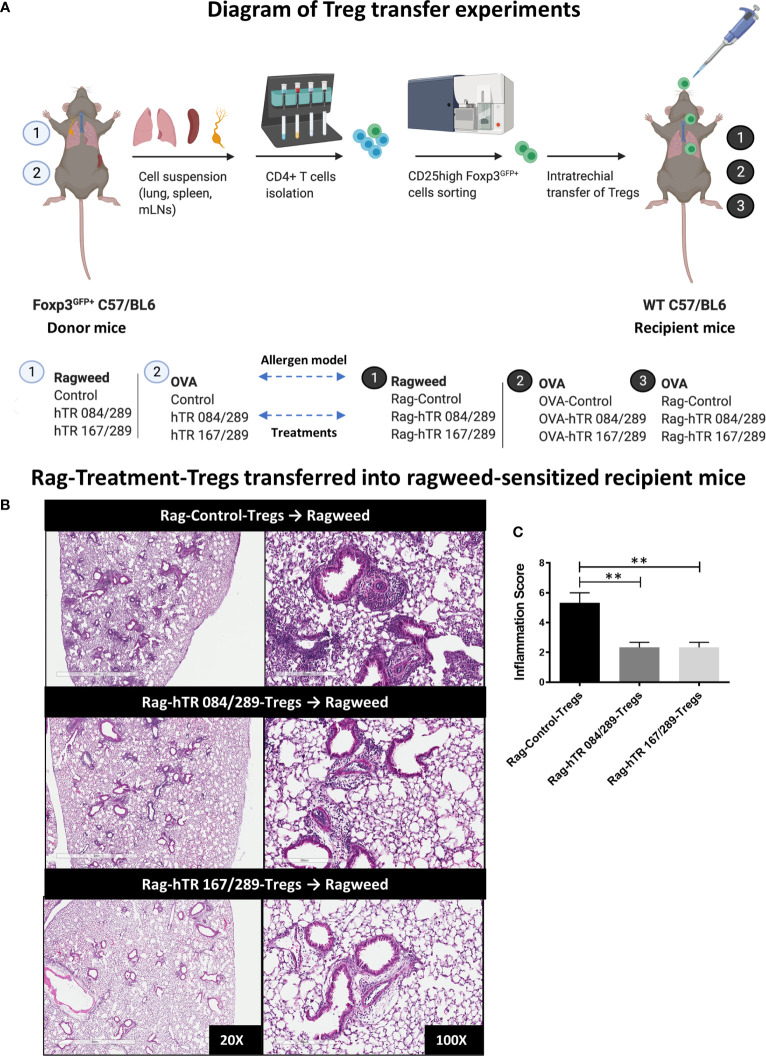
Tregitopes-Tregs are more efficient at improving murine airways disease than Control-Tregs *in vivo*. **(A)** Diagram depicting the Treg transfer experiment used to determine the efficacy and antigen-specificity of Tregs from Tregitopes treated allergic mice. Donor mice were sensitized with either Ragweed or OVA and treated with Control and Tregitope combinations as indicated (① or ②). Treg (CD25highFoxP3-EGFP+) cells were isolated from lungs, spleen and mediastinal lymph nodes of donor mice and transferred to recipient mice. Sensitization with antigen primed-BMDCs in recipient mice was performed as described in *Methods*. On day 9 after sensitization, Control Treg or Antigen-Specific Tregs were adoptively transferred to the lungs of sensitized recipient mice as indicated followed by antigen challenge on day 10. **(B)** Ragweed-sensitized recipient mice were treated with 50,000 Control Treg, Rag-hTR 084/289-Tregs or Rag-hTR 167/289-Tregs derived Treg ➊, then challenged with Ragweed, and lungs were harvested after 72 hours, H&E-stained lung sections were examined by microscopy (20X, 100X) **(C)** and scored for inflammation by two independent observers. Representative of 2 independent experiments, n = 3-4. **p, 0.01, one-way ANOVA test.

We first performed a Treg dose-response experiment to determine the absolute number of Ragweed-Control-Tregs that could be adoptively transferred without abrogating the lung inflammatory responses in a non-specific manner. To perform the dose-response study, we induced AAD by intratracheally transferring ragweed-treated BMDCs in the lungs of mice followed by antigen challenge 10 days post transfer (**Figure 5A**). Adoptively transferring 200,000 Ragweed-Control-Tregs (not treated with Tregitope) prior to challenge, was effective at ameliorating airway inflammation in a non-specific manner (**Supplemental Figure S2**). In contrast, transferring a lower dose of 50,000 Ragweed-Control-Tregs did not rescue inflammation, making it possible to contrast this dose of non-specific Tregs to Tregitope-and-allergen-treated Tregs on lung inflammation (**Supplemental Figure S2**). Adoptively transferring 50,000 Ragweed and hTregitope 084/289-treated Tregs and Ragweed and hTregitope 167/289-treated Tregs prior to challenge reduced airway inflammation as demonstrated by histology and inflammation scores, compared to the Tregs not treated with Tregitopes and allergen control group (**Figures 5B, C** **)**.

**Figure 6:**
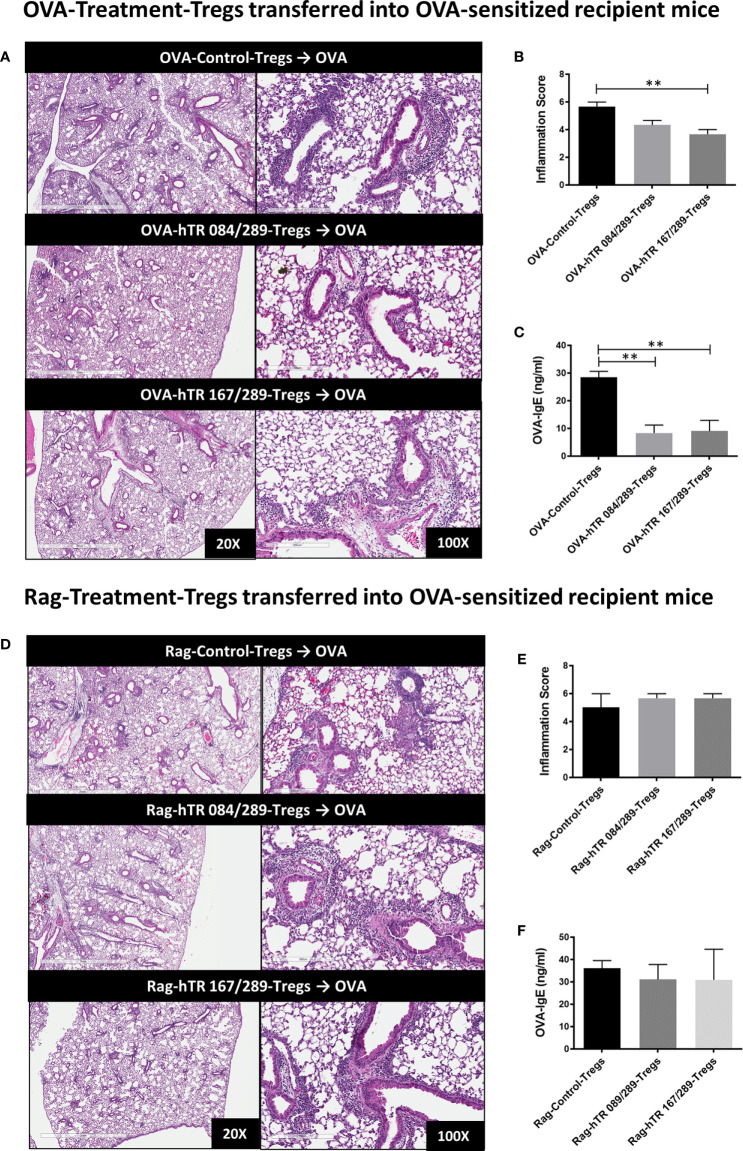
Tregitopes induce antigen-specific Tregs. **(A)** Please see schematic in **Figure 5A**. OVA sensitized mice were treated with 50,000 OVA-Control-Tregs, OVA-hTR 084/289-Tregs or OVA-hTR 167/289 -Tregs ➋, challenged with OVA, and lungs were harvested after 72 hours. H&E-stained lung sections were examined by microscopy (20X, 100X) **(B)** and scored for inflammation by two independent observers. **(C)** OVA-specific plasma IgE levels were significantly diminished in OVA-Treg transferred mice. **(D)** OVA sensitized mice were treated with 50,000 Rag-Control-Tregs, Rag-hTR 084/289-Tregs or Rag-hTR 167/289 -Tregs ➌, challenged with OVA, and lungs were harvested after 72 hours. H&E stained lung sections were examined by microscopy (20X, 100X) **(E)** and scored for inflammation by two independent observers. **(F)** In contrast to panel **(C)**, OVA-specific plasma IgE levels were not diminished in Rag-Treg transferred mice. Representative of 2 independent experiments, n = 3-4 **p, 0.01, one-way ANOVA.

Both OVA and hTregitope 084/289-treated Tregs (OVA-hTR 084/289-Treg) and OVA and hTregitope 167/289-treated Tregs (OVA-hTR 167/289-Treg) improved airway inflammation when transferred to OVA-allergic mice and were significantly more suppressive than OVA and vehicle-control-treated Tregs (OVA-Control-Tregs) as shown by significant decreases in OVA-specific IgE and lung inflammation scores (**Figures 6A–C**). This replicates the data obtained, showing that Ragweed-Tregitope-Tregs improve ragweed-driven lung inflammation more effectively than Ragweed-vehicle control-treated Tregs (**Figures 5B, C** **)**. We then determined if Tregitope-induced Tregs required antigen-specificity to be effective by transferring 50,000 Ragweed-Tregitope-Tregs to OVA-sensitized mice prior to challenge. At a dose of 50,000 Tregs per lung, Ragweed-hTregitope 084/289-treated Tregs (Rag-hTR 084/289-Treg), and Ragweed-hTregitope 167/289-treated Tregs (Rag-hTR 167/289-Treg) did not ameliorate lung inflammation with inflammation scores in OVA-sensitized mice, and did not suppress OVA-specific IgE levels across all Tregitope treatment groups (**Figures 6D–F**). These transfer studies strongly suggest that Tregitope treatment induced antigen-specific Tregs.

## Discussion

In this study, we have carefully dissected the regulatory responses involved in the anti-inflammatory activity of Tregitopes using murine models of allergic airways disease (AAD). We showed that treatment with murine (m) and human IgG Tregitopes (hTR) alleviated allergen-induced-airway hyper-responsiveness and reduced lung inflammation. The action of the human IgG Tregitopes attenuated allergic airways disease (in mice) in a manner similar to that of IVIg. However, in addition to increases in peripheral CD25^high^ Tregs in mediastinal lymph nodes (mLN), there was an expansion of Helios+ Tregs upon Tregitope treatment. Importantly, Tregs from IgG Tregitope-treated mice were demonstrated to be more highly suppressive compared to similar numbers of Treg derived from controls. Furthermore, when Tregs from IgG Tregitope-treated mice were transferred into allergen-sensitized mice, we demonstrated that only Tregitope-induced Tregs from mice exposed to the same allergen were capable of attenuating AAD. The antigen-specificity of the regulatory T cell response in the Tregitope-allergen treatments has important implications for safety and specificity in clinical translation.

We have previously demonstrated that human IgG Tregitopes bind to murine MHC (12) and therefore employed two different formulations of human Tregitopes in this study: hTregitopes 167/289, and hTregitopes 084/289. Although both formulations were efficient at diminishing lung neutrophilia, IL-13 concentrations, IL13+CD4+ T cells, and increased peripheral Tregs, hTregitopes 167/289 abrogated IL-17A production by CD4+ T cells in both OVA and ragweed models while hTregitopes 084/289 was effective in the ragweed model only. Lung cell counts were also decreased in allergic mice, following treatment with hTregitopes 167/289 to an extent greater than with hTregitopes 084/289 (**Supplemental Figure S4**). The impact of hTregitopes 167/289 on lung neutrophils has implications for other neutrophil-mediated pulmonary diseases.

T regulatory cells play a significant role in establishing airway tolerance. Treatments like IVIg, capable of inducing highly suppressive total and *de novo* Tregs in an antigen-specific manner, can improve AAD in mice (5, 14). In this study, we showed similar results. Total Tregs from Tregitopes-treated mice had higher suppressive capacity compared to vehicle control. Although at high enough doses, Tregs can inhibit inflammatory responses non-specifically, by using titration studies, we demonstrated that it took 4-fold less CD4+FoxP3+ Tregs from Tregitope-treated mice to attenuate the inflammatory changes observed following antigen challenge. This observation has two potential explanations. IgG Tregitope-induced T-reg may have properties that increase their stability and diminish their plasticity leading to a more suppressive phenotype, or IgG Tregitope treatment may induce antigen-specific Tregs, enabling them to interact with APC at the site of antigen presentation and suppress the action of effector T-cells.

Consistent with both hypotheses, IgG Tregitope treatment of mice in the context of allergen led to an increase in mLN Tregs which was most prominent in CD4+Helios+FoxP3+ cellular subset. Helios+ Tregs are associated with Treg cells that are highly suppressive and have less plasticity (20, 21).

Initially, Helios+ Tregs were only found in nTreg of thymic origin (22, 23). This may have been due to the conditions for induction of the Treg. Indeed, Gottschalk et al. showed that pTreg could be Helios+ if the conditions for induction of Treg included antigen presenting cells and sources of antigen (20, 21, 24). This is very relevant for our whole animal AAD model, which required APCs and antigen for the induction of Treg by IVIg (15). This finding from our current study may indicate a key advantage for an IgG Tregitope-based immunotherapy with potential to accelerate and maintain stable allergen-specific tolerance.

Previous work in *in vitro* assays using human PBMCs from individuals with birch-pollen allergy, where the allergen was co-incubated with either of the IgG Fc-derived hTregitopes,167 or 289, revealed a shift from Th2 to Th1/Treg response and showed that Tregs that were induced after 30 days of Tregitope treatment *in vitro* were specific to the Birch-pollen antigen (7). Antigen-specificity has also been demonstrated in several transplant models (25). The studies performed herein confirmed the potency of these IgG-derived Tregitopes in AAD and separately, the adoptive transfer studies corroborated the ability of Tregitopes to induce antigen-specific Tregs in the allergic asthma model. Tregitope treatment in conjunction with allergen immune tolerance induction may have the potential to contribute to a more stable Treg Helios+ phenotype. Further characterization of Treg cells with antigen-specific tetramer would provide conclusive evidence that the Helios+ Tregs are antigen-induced iTregs.

We observed subtle differences between the two combinations of the IgG Tregitopes that we employed in these studies, hTregitope 167 or hTregitope 084 in combination with hTregitope 289. The variability in efficacy may be explained by intrinsic differences, in nature and function, of the peptides such as binding affinity for different MHC molecules in the murine model. EpiMatrix-predicted MHC binding promiscuity (EMX) score for the C57BL/6 MHCII, which parallels the T cell response, amounts to 10.67 and 1.94 for hTregitope 167 and hTregitope 084, respectively (**Supplemental Table S1**) (6, 7, 26). Additionally, we expect that epitopes that are cross-conserved to other self-proteins on the TCR face to be more tolerogenic, and evaluation using JanusMatrix reveals that hTregitope 167 has 24 TCR matches in the murine genome, as compared to 6 for hTregitope 084 (27) (See **Supplemental Table S1**). Alternatively, one or another formulation of Tregitopes may be more efficient depending on the specific disease model. Furthermore, given the different amino acid sequences and resulting specificities of each peptide, peptide stability may differ.

We note that the effect of Tregitopes has been described extensively in both *in vitro* studies using human T cells as well as in animal models. Despite these findings, potential alternative explanations for the effect of Tregitopes have been suggested. Studies that compared their effect on suppressing CD8+ T cell restricted immune responses have dispelled the concept that the effect is due to competition for HLA binding. Careful use of control arms in *in vivo* models, as described here, show that the effect is not due to small amounts of DMSO that is present in the solution used to dissolve the Tregitope peptides. Peptides are not the only means of delivering Tregitopes: Tregitopes are also effective when fused with albumin (8, 19), delivered in AAV as a transgene (28), and when conjugated to a carrier protein. IgG Tregitopes are active in TCR-transgenic mice, but not when TCR recombination is inhibited, suggesting that IgG-Tregitope-specific T cells may themselves be Tregitope specific. The Tregitope effect requires direct contact between Tregs and T-effectors *in vitro* (29), suggesting that while cytokines signal their effect, they are not required for the immunomodulatory properties of Tregitopes. This study further elucidates the expected effect of Tregitopes, which is to transform antigen-specific T cells into antigen-specific Tregs resulting in a highly specific immunomodulatory therapy that has potentially protective effects on lung inflammation. In concurrence with previously published data in NOD mice, here we have shown that Tregitope treatment with antigen produces antigen-specific Tregs, which can suppress reactivity in similar antigen-challenged mice as shown by these adoptive transfer experiments.

In this study, Tregitopes exhibited tolerizing effects similar to the IVIg treatment groups in both the OVA and ragweed allergy models. We postulate that Tregitopes are among the active components (similar to the active pharmaceutical ingredient, or API) within IVIg, and can be considered to be one of the mechanisms by which IVIg therapy is effective at ameliorating inflammatory response (12, 30). As demonstrated in a clinical trial, the use of high dose intra-venous immunoglobulin (IVIg) can improve severe asthmatic condition in children, an observation that has been reproduced in adults (31, 32). IVIg is more commonly used for organ transplant recipients and autoimmune diseases (33–35) due to the need to infuse IVIg and low patient acceptance of IVIg due to adverse effects during infusion. IVIg is a costly human blood-derived product that also presents potential risks associated with adverse reactions including renal impairment, thrombosis and hemolytic anemia (36). Tregitope-based therapy may lead to improved treatments for allergy and other inflammatory conditions, over IVIg. IgG Tregitope-based treatment could become a synthetic alternative to IVIg, and unlike steroid-based treatments for allergic asthma, is not a general immunosuppressant.

In conclusion, Tregitopes, in combination with allergen, provides a natural mode of immune tolerance that may induce highly suppressive Helios+ Treg in an antigen-specific manner. Tregitope-based immunomodulation holds promise as a novel treatment to reduce reactive airway disease in humans and other allergic conditions.

## Data Availability Statement

All relevant data generated for this study are included in the article and the **Supplementary Material**. The raw data supporting the conclusions of this manuscript will be made available by the authors to any qualified researcher upon request.

## Ethics Statement

The animal study was reviewed and approved by McGill University Animal Care Committee and are in accordance with the guidelines of the Canadian Council on Animal Care (CCAC).

## Author Contributions

MD, BM, and ADG conceptualized and designed the study and experiments. Experiments were performed by MD, AM, and ST. Data were analyzed by MD, AM, and BM. MD, ADG, SM, SL, and BM were involved in drafting and or revising the manuscript. All authors contributed to the article and approved the submitted version.

## Funding

The studies were supported by grants from the Canadian Institutes for Health Research (CIHR ID 246796), and the Montreal Children’s Hospital Foundation. BM was a recipient of a Chercheur Nationale Award from the Fonds de Recherche en Sante- Quebec.

## Conflict of Interest

AG is a senior officer and shareholder, and SM and SL are employees of EpiVax, Inc., a company specializing in immunoinformatic analysis. EpiVax, Inc. own patents to technologies utilized by associated authors in the research reported here. BM holds research funds from Canadian Institutes for Health Research, National Sciences Engineering and Research Council, The McGill University Health Center Foundation and The Montreal Children’s Hospital Foundation.

The remaining authors declare that the research was conducted in the absence of any commercial or financial relationships that could be construed as a potential conflict of interest.

## Abbreviations

Tregitope, Regulatory T Cell epitope in IgG; AAD, Allergic Airways Disease; AHR, Airway Hyperresponsiveness; BAL, Bronchoalveolar lavage fluid; Tregs, Regulatory T Cells; iTregs, induced Regulatory T Cells; nTregs, natural Regulatory T cells (thymic-derived); pTregs; peripheral Regulatory T Cells; DC, Dendritic Cells; MHC, Major Histocompatibility Complex; TCR, T Cell Receptor; BMDC, Bone Marrow-derived Dendritic Cells; H&E staining, Hematoxylin and eosin stain.
