# Supplementary material for: Tregitopes Improve Asthma by Promoting Highly Suppressive and Antigen-Specific Tregs
Source: Front Immunol. 2021 Apr 19;12:634509. doi: 10.3389/fimmu.2021.634509 (PMC8089381; doi:10.3389/fimmu.2021.634509)
Supplement: Supplementary file 1 [file DataSheet_1.pdf]

Supplementary Material

Peptide ID	Abbrev. Name	Source	Peptide Sequence	H2-I-Ab EpiMatrix max EPX*	H2-I-Ab EpiMatrix cluster EPX*	EPX* hits	JMX* hits
mTregitope 167	mTR 167	IgG1 (CH)	PAVLQSDLYTLSSSVTVPSSTWPSQ	2.48	8.42	4	37
hTregitope 167	hTR 167	IgG1 (CH)	PAVLQSSGLYSLSSVVTVPSSSLGTQ	2.61	10.67	5	24
hTregitope 289	hTR 289	IgG1 (CH)	EEQYNSTYRVVSVLTVLHQDW	2.58	6.72	3	3
hTregitope 084	hTR 084	IgK (VL)	GTDFTLTISSLQPED	1.94	1.94	1	6

**Abbreviations: EPX, EpiMatrix; JMX, JanusMatrix; Max EPX, Maximum EpiMatrix Score; Cluster EPX, Cluster EpiMatrix Score.*

Supplemental Table S1. Sequences of the Tregitopes used in current study are shown with their EpiMatrix (EPX) prediction z-scores and total hit counts for C57Bl/6 mice. EpiMatrix is a T cell prediction tool that calculates binding probability scores (1). An EPX z-score > 1.64 or more is considered a "hit", meaning that these epitope frames have a significant chance of binding to C57BL/6 MHC molecules with moderate to high affinity, and therefore, have a good chance of being presented on the surface of APCs. The EpiMatrix Cluster Score is a sum of the probability scores across the entire peptide sequence, whereas the Maximum EpiMatrix Score (Max EPX) refers to the maximum score within the peptide, i.e. for the 9-mer with the highest binding likelihood. JanusMatrix hits indicates the number of murine protein matches with the exact T cell receptor (TCR) facing residues within the predicted 9-mer MHC binding frame, and it is a measure of cross-conservation and potential occurrence of T cells that share the same TCR-facing residues (2). JanusMatrix hits may indicates that many homologous MHC binding peptides are found within the mouse proteome, suggesting that the epitope may be tolerated or potentially tolerogenic. Note that there are 4 MHC binding 'hits' in the mTregitope 167 sequence which has a maximum EPX z-score of 2.48 (top 1%) and many matches to TCR-facing residues in the mouse proteome (37 JMX hits). Similarly, there are 5 murine MHC motifs (EPX hits) in hTregitope 167 and the maximum EPX z-score is 2.61 (top 1%). hTregitope 167 also has several matches to other mouse protein epitopes at the TCR face as determined by JanusMatrix (JMX hits) but not as many as murine Tregitope 167. Both hTregitope 167 and mTregitope 167 are located in the CH1 domain of Fc. hTregitope 289, located in the CH2 Fc domain, has 3 murine MHC motifs with a maximum EPX z-score of 2.58, and 3 cross-conserved hits in the murine proteome. hTregitope 084 is derived from the kappa variable region and also has a high EPX z-score for murine MHC (1.64, top 5%) but it contains only one predicted binding motif.

1. Schafer J. Prediction of well-conserved HIV-1 ligands using a matrix-based algorithm, EpiMatrix. *Vaccine* (1998) **16**:1880–1884. doi:10.1016/S0264-410X(98)00173-X

2. Moise L, Gutierrez AH, Bailey-Kellogg C, Terry F, Leng Q, Abdel Hady KM, VerBerkmoes NC, Sztein MB, Losikoff PT, Martin WD, et al. The two-faced T cell epitope: examining the host-microbe interface with JanusMatrix. *Hum Vaccin Immunother* (2013) **9**:1577–86. doi:10.4161/hv.24615

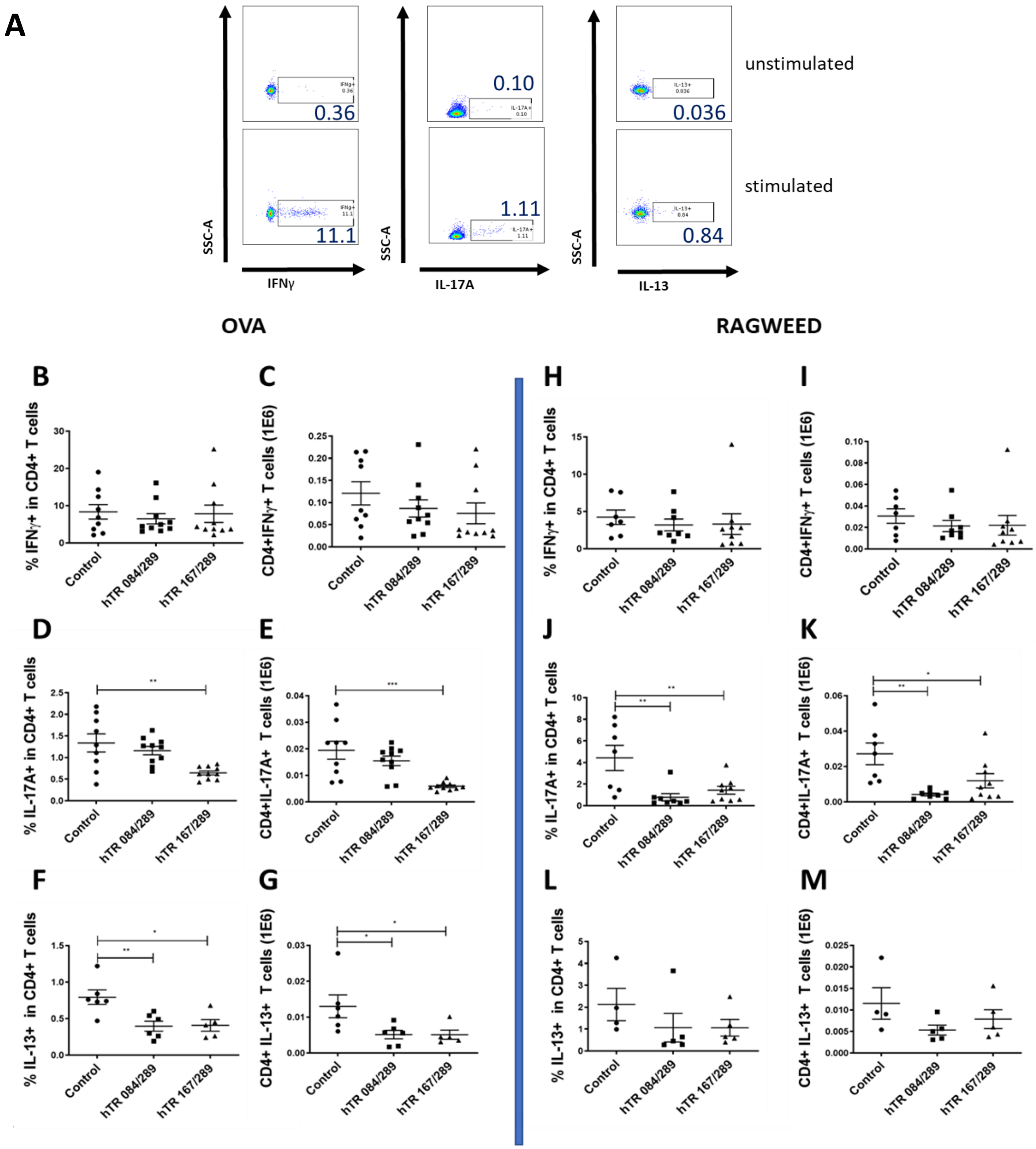

Supplemental Figure S1. Tregitopes modulate cytokine expression in lung T effector cells. T cells were extracted from gently homogenized lungs of Tregitope and IVIG treated mice and stained for intracellular cytokine expression prior to flow cytometry. (A) Lung cells were stimulated with PMA and inonomycin for 4 hours, then the samples stained with intracellular markers and gated for intracellular IFNγ, IL-17A and IL-13 expression in CD4+ T cells. We use an unstimulated sample to help us gate for positive cytokine expression. Percentage of CD4+ T cells expressing IFNγ, IL-17A, IL-13 in OVA and ragweed allergic mice (B, D, F, H, J, L). Absolute number of CD4+ T cells expressing IFNγ, IL-17A and IL13 cells in whole lung (C, E, G, I, K, M). ***p, 0.005, **p, 0.01, *p, 0.05, one-way ANOVA test.

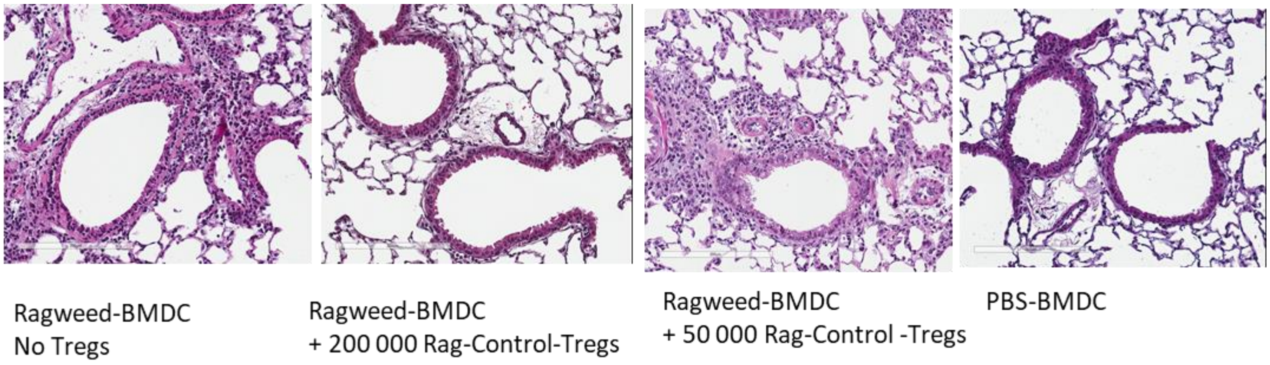

Supplemental Figure S2. Dose response effect of Vehicle Control-Tregs on allergic inflammation. Inflammatory infiltration of allergic airway disease mice is reduced by adoptive transfer of high doses of non-specific Treg but not by lower doses of non-specific Tregs. The lower dose was selected for comparison with antigen-specific Tregs. Scanned H&E stained lung sections of allergic mice (ragweed-BMDCs) receiving No Tregs, 200 000 Rag-Control-Tregs and 50 000 Rag-Control-Tregs.

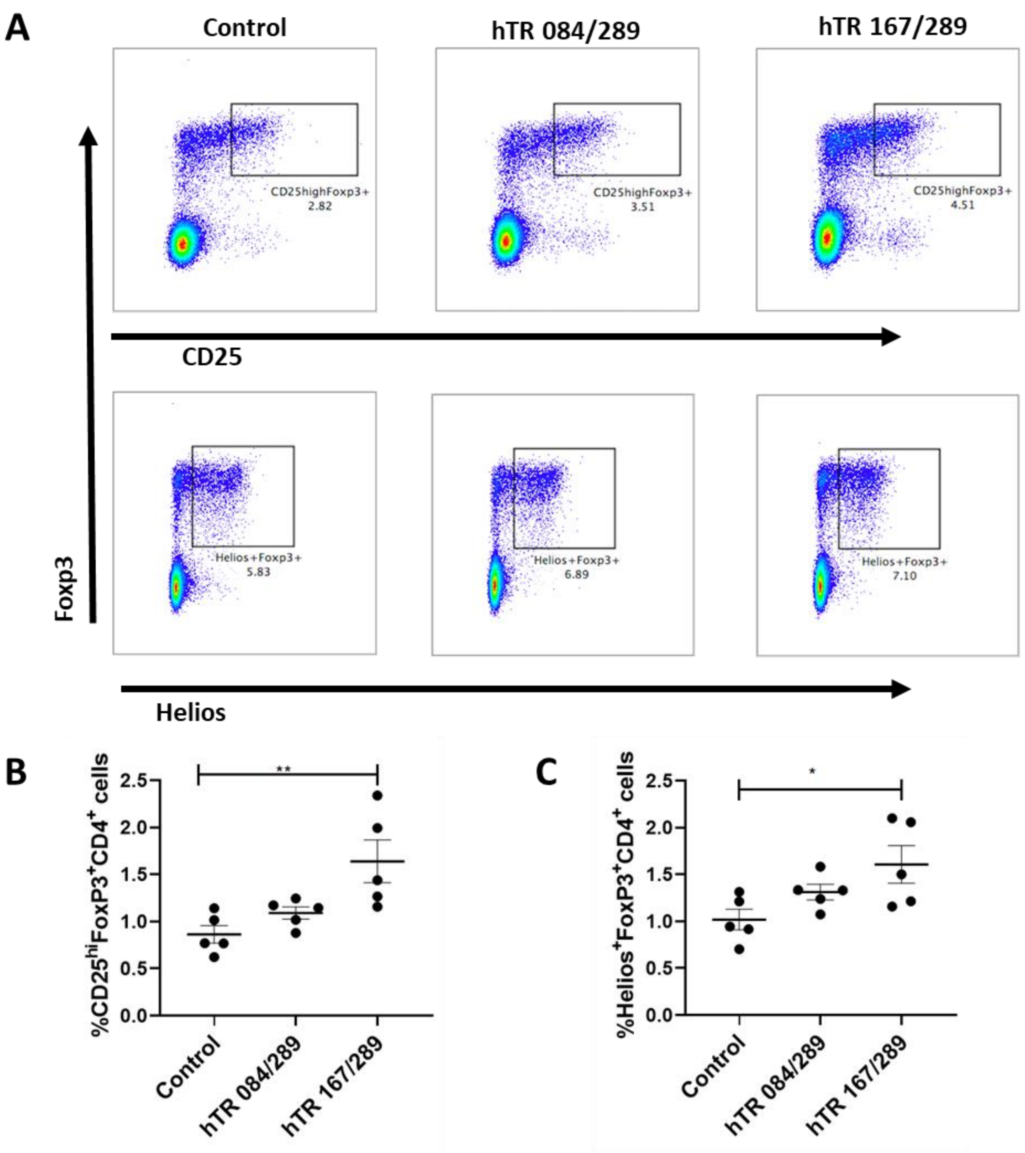

Supplemental Figure S3. Tregitope treatment expand CD25+ Tregs and Helios+ Tregs in LNs of ragweed-allergic mice. LNs cell suspension from ragweed-allergic mice treated with Control, hTR 084/289 or hTR 167/289 were processed and stained for Tregs. (A) Gating strategy for Treg phenotyping. Percentage of (B) $CD25^{hi}$Foxp3+ Tregs and (C) Helios+Foxp3+ Tregs in LNs of ragweed allergic mice. *p, 0.05, **p, 0.01, one-way ANOVA test.

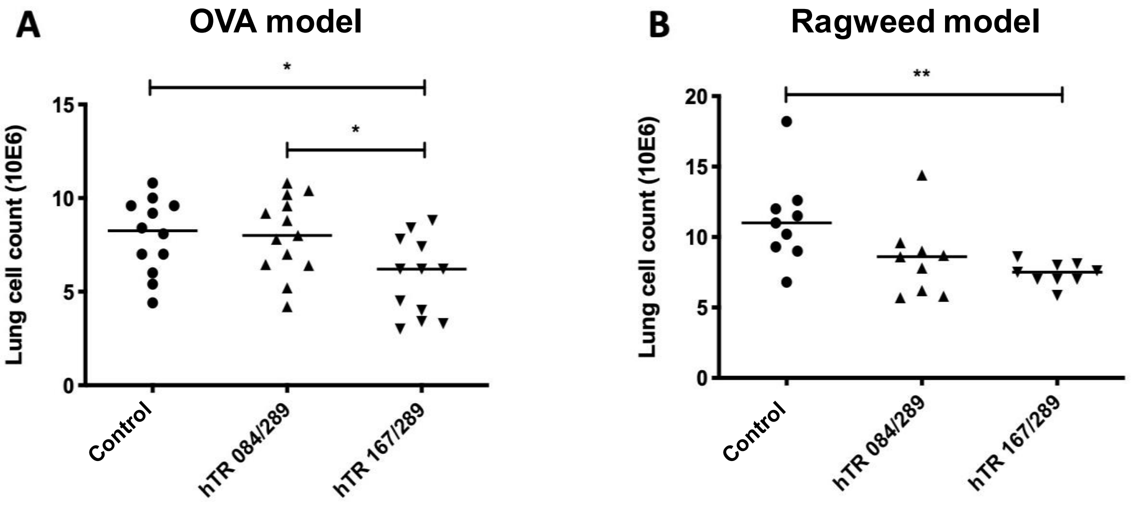

Supplemental Figure S4. Comparison of total cell counts in two AAD models. The total cell counts for lungs of mice induced to be allergic to OVA (A) and for lungs of mice induced to be allergic to Ragweed (B) are shown. Lung cell counts were performed using a Beckman Coulter counter.
